# Alcohol use in a rural district in Uganda: findings from community-based and facility-based cross-sectional studies

**DOI:** 10.1186/s13033-018-0191-5

**Published:** 2018-04-03

**Authors:** Oliva Nalwadda, Sujit D. Rathod, Juliet Nakku, Crick Lund, Martin Prince, Fred Kigozi

**Affiliations:** 1 Butabika National Referral Mental Hospital, P.O Box 7017, Kampala, Uganda; 2 London School of Hygiene and Tropical Medicine, London, UK; 3 Alan J Fisher Centre for Public Mental Health, Department of Psychiatry and Mental Health, University of Cape Town, Cape Town, South Africa; 4 Institute of Psychiatry, Psychology and Neuroscience, King’s College, London, UK

**Keywords:** Alcohol use, AUDIT, Internalized stigma, Help-seeking, Men, Uganda

## Abstract

**Background:**

Uganda has one of the highest per capita alcohol consumption rates in sub-Saharan Africa. However, the prevalence of alcohol use disorders (AUD) remains unknown in many areas, especially in rural districts. This study aimed to estimate the prevalence of alcohol consumption and of alcohol use disorder among men, and to describe the distribution of drinking intensity, among men in in Kamuli District, Uganda.

**Methods:**

Men attending primary care clinics in Kamuli District were consecutively interviewed in a facility-based cross-sectional study, and a separate group of men were interviewed in a population-based cross-sectional study. In both studies the men were administered a structured questionnaire, which included the alcohol use disorder identification test (AUDIT) to screen for AUD, as well as sections about demographic characteristics, depression screening, internalized stigma for alcohol problems and treatment-seeking.

**Results:**

Among the 351 men enrolled in the Community study, 21.8% consumed alcohol in the past 12 months, compared to 39.6% of 778 men in the Facility Survey. The proportion of men who screened positive for AUD was 4.1% in the community study and 5.8% in the facility study. AUDIT scores were higher among older men, men with paid/self-employment status and higher PHQ-9 score (P < 0.05). Nearly half (47.5%) of the men with AUDIT-positive scores reported that alcohol use problems had ruined their lives. A majority (55.0%) of men with AUDIT-positive scores did not seek treatment because they did not think AUD was a problem that could be treated.

**Conclusions:**

Internalized stigma beliefs among AUDIT-positive men impede treatment-seeking. As part of any efforts to increase detection and treatment services for alcohol use problems, routine screening and brief interventions for internalized stigma must be incorporated within the normal clinical routine of primary health care.

**Electronic supplementary material:**

The online version of this article (10.1186/s13033-018-0191-5) contains supplementary material, which is available to authorized users.

## Background

Alcohol use disorders (AUD) are characterized by harmful, hazardous or dependent patterns of alcohol consumption that result in adverse physical and mental health consequences. Alcohol is a causal factor in 60 types of diseases and injuries and a component cause in 200 others [1]. It accounts for 4% of the global burden of disease [2, 3] and the global net effect of alcohol consumption on health is estimated at 3.8% of all global deaths [1]. According to the Ugandan Ministry of Health, alcohol dependency is among the main causes of psychiatric morbidity in Uganda [4]. AUD is also associated with many serious social issues, including violence, child neglect and abuse, and absenteeism in the workplace [1, 5]. In addition, AUD negatively impacts economic wellbeing through decreased work productivity, impaired role performance and increased expenditure on alcoholic beverages [5–7] resulting in various forms of social disadvantage such as stigmatization [8].

Men in Uganda are estimated to have one of the highest alcohol per capita consumption levels in sub-Saharan Africa with 25.6 litres of pure alcohol consumed among males each year [1, 9]. The 12 month prevalence of AUD in Uganda is estimated at 10% among males [1] and the prevalence of alcohol dependence is estimated at 4.2% among males [1]. To date, no study has examined the prevalence and characteristics of men who drink in eastern Uganda, specifically in Kamuli district. Previous studies in Uganda were mainly conducted in specialised sub-populations such as fishing communities [10], post-conflict areas [11], HIV patients [12–14] and university students [15, 16]. These findings may not be applicable to regions like Kamuli that have no university or any higher institution of learning, and are neither post-conflict areas nor predominantly fishing communities. Kamuli is however similar to many other parts of Uganda, and the findings from this study will be more generalizable to other predominantly rural communities unlike those from previous studies. Given the paucity of research, the current study sought to estimate the prevalence of alcohol consumption and of alcohol use disorder among men, and to describe the distribution of drinking intensity, among men in Kamuli District, Uganda. To inform planning of health promotion activities and service delivery, we have characterized community- and facility-based samples of men who drink and described their internalized stigma beliefs as well as attitudes and beliefs towards help seeking.”

## Methods

### Setting

This study was conducted as part of the PRogramme for Improving Mental health carE (PRIME), which aims to reduce the treatment gap for mental disorders by implementing mental health care plans for depression, AUD, psychosis and epilepsy in the primary health care settings in five low and middle income countries including Kamuli District, Uganda [17]. Kamuli district has a total population of 490,255 of whom 48% are males [18]. The majority (96%) of the population resides in rural areas and 58% live in poverty [18]. The district has one government hospital, one non-profit hospital and 24 government health centres at various levels. Mental health services including counselling and medication are only available at the government hospital [19]. At the time of this study, inpatient detoxification and rehabilitation services were not available in Kamuli, however, rehabilitation services were available at Jinja main hospital which is approximately 64 km away from Kamuli district. A situational analysis conducted in the district [20] revealed that the majority of people with alcohol related problems prefer to seek care from the regional or national referral hospitals, which are located 64 and 155 km from the district centre, respectively. Kamuli was chosen for the integration of mental health services into primary health care as a representative of the wider socio-cultural, and under resourced economic contexts in Uganda offering opportunities for adaptation of interventions and generalization of the research findings in other diverse socially disadvantaged populations within Uganda. Full details of the country sites are presented elsewhere [21].

### Community study

A detailed description of the Community study protocol, including sampling plan, sample size calculation, and questionnaire design has been previously described [22]. Briefly, between May and August 2013, 25–60 households were randomly selected per village from 30 villages. At each selected household, the interviewers located an adult member, explained the aims of the study and asked that member to identify all adults who were available to be interviewed in that household. A random selection of one adult was made who was then assessed for eligibility and then recruited into the study; a total of 1291 adults were approached and 1290 (99.9%) consented to participate in the study. The eligibility criteria included being adult (≥ 18 years of age), fluent in English or the local language (Luganda) and those willing/able to complete the full interview. In a private location selected by the participant, the interviewer administered the paper questionnaire. The questionnaire had two parts: Part 1 had sections on basic socio demographic characteristics, depression, and alcohol use, Part 2 had sections on detailed demographic characteristics, economic status, internalised stigma as well as attitudes and beliefs towards help seeking. Only participants who screened positive for depression or for AUD completed Part 2. Finally, a research assistant entered the response data into a questionnaire application programmed on an Android device.

Before enrolment into the study, the eligible participants were informed about the aims of the study, the reason for their selection, potential length of the interview, the collaborating partners involved in the research and their discretion to participate or withdraw at any time during the study. Participants were assured that all information obtained from them would be kept confidential. Written informed consent in the form of a signature or thumbprint was obtained from all participants.

### Facility study

A facility survey was conducted between July and November 2013. Participants were consecutively recruited from 13 primary care clinics within Kamuli District. Every respondent meeting the criteria of inclusion was interviewed until the required sample size of 1800 was achieved; a total of 1922 adults were approached and 1893 (98.5%) consented to participate in the study. The sample size of 1800 respondents was distributed in proportion to each clinic’s average patient volume in December 2012 which ranged from 362 in a level 5 clinic (equivalent to the outpatient department in the district hospital) to 66 in a level 3 clinic (equivalent to a community primary care centre). The eligibility criteria, informed consent process and data collection for the facility study are largely identical to the Community study. Exceptions were that patients were ineligible if they required acute medical care that the interview and data collection was conducted on an Android mobile device.

### Study questionnaires

The questionnaire sections in the two studies were largely identical. For the socio-demographic and economic status questions, we adapted questions from the Uganda Demographic and Health Surveys (2006) [4].

To screen for AUD, the 10-item Alcohol Use Disorders Identification Test (AUDIT) was used [23]. Each item concerns the frequency of different drinking behaviours and consequences over the past 12 months. Responses are scored from 0 (Never occurs) to 4 (Daily). The sum of scores indicate whether the respondent engages in hazardous (score 8–15), harmful (score 16–19) or dependent (score ≥ 20) drinking behaviours, using cut-offs defined by the World Health Organization [24, 25]. For this study, a score of 8 or more was considered to be a positive screening result. The AUDIT was adapted for local use through the use of pictures and local terms for standard alcohol units. An additional translated AUDIT file shows this in more detail (see Additional file 1). For men, the Cronbach’s alpha for the AUDIT was 0.76.

To assess for depression, the 9-item Patient’s Health Questionnaire (PHQ-9) was used [25]. The nine items of the PHQ-9 are based directly on the nine diagnostic criteria for major depressive disorder in the DSM-IV [26]. Each item is scored on a Likert scale with symptoms rated as 0 (not at all), 1 (several days), 2 (more than half the days) and 3 (nearly every day). The sum of the scores indicates whether the respondent has mild depression (score 5–9), moderate depression (score 10–14) or severe depression (score ≥ 15). For men, the Cronbach’s alpha for the PHQ-9 was 0.80.

To assess internalised stigma among AUDIT-positive men, the Internalized Stigma of Mental Illness scale [27] was used. Eleven questions from the 29-item Internalized Stigma of Mental Illness scale with regard to “problems with drinking” were asked. These items were selected for their relevance across all PRIME settings.

The attitudes and barriers to health care seeking were examined using the Barriers to Access to Care Evaluation scale (BACE) [28]. This 24-item scale comprises of a comprehensive list of key barriers to access to mental health care with categorical responses (i.e. “strongly agree,” “agree,” “neutral,” “strongly disagree” and “disagree”). The instructions were adapted in the study to address barriers to care for alcohol use.

### Statistical analysis

First, the demographic characteristics (i.e. age, educational attainment, employment status) were described as well as the PHQ-9 scores and AUDIT scores of men in the community (n = 351) and facility surveys (n = 778). For this step of the analysis, the Community study figures were adjusted for the complex sampling design (i.e. strata, clusters and probability weights).

Second, the community and facility study samples of men who drink were combined to evaluate whether the distribution of AUDIT scores among men who drink varied by socio-demographic characteristics, PHQ9 score or study base (i.e. community or facility). As the distribution of AUDIT scores was skewed we could not use linear regression to test for associations. Instead, we report the median and interquartile range for each stratum and used the Kruskal–Wallis test to test for an association with the AUDIT score. We repeated the associations analysis with a binary AUDIT score using Fisher’s exact test, and reported the proportion of participants in each category who screen positive (AUDIT score ≥ 8). Due to the small employment strata, people with voluntary employment were combined with the unemployed, and retired with other. We used age categories of < 30 years, 30–44, 45–59 and ≥ 60 as per the Uganda Demographic and Health Study [4].

Third, we describe the internalised stigma-related beliefs for men who screen AUDIT positive in the Community Survey. For reporting these descriptive statistics, we combined those who said they “agree” or “strongly agree” with each statement and adjusted the percentage figures for the complex sampling design. For AUDIT positive men in the Facility Survey, we describe the attitudes and beliefs towards help seeking.

We completed all analyses in Stata SE 14.2 (College Station, TX, USA).

## Results

### Socio-demographic characteristics

The demographic, depression and alcohol-related characteristics of men in the Community Survey and Facility Survey are reported in Table 1.

**Table 1:** Demographic, depression and alcohol consumption characteristics of men in Kamuli District, Uganda, 2013

Characteristics	Community survey (n = 351)	Facility survey (n = 778)
Category		
Age, years (%)		
< 30	32.9	33.1
30–44	36.7	34.8
45–59	21.0	22.5
≥ 60	9.4	9.6
Education attainment (%)		
Less than primary education/illiterate	9.4	8.3
Completed primary education	48.3	45.0
Completed secondary education	35.6	33.9
Completed university diploma/degree	6.7	12.8
Employment status (%)		
Paid or self-employment	60.2	54.1
Unemployed or volunteer	26.3	11.1
Student	9.3	10.5
Retired	4.2	4.1
Other	0	20.2
Depression category (%)		
None	72.4	74.9
Mild	23.8	19.4
Moderate	3.2	5.4
Severe	0.6	0.3
Alcohol drinking category (%)		
Non-drinker	78.2	60.4
Non-hazardous	17.7	33.9
Hazardous	2.9	4.5
Harmful	0.7	0.6
Dependent	0.5	0.6

In the Community Survey, more than one-third (36.7%) were aged 30–44 years of age. Almost half (48.3%) of the men had primary education while majority (60.2%) of the men had paid or self- employment status. In the Facility Survey, approximately one-third (34.8%) were aged 30–44 years. Over four in every ten men (45.0%) had primary education while slightly over half (54.1%) of the men had paid or self-employment status.

In the Community study, 21.8% of men had consumed alcohol in the past year and 4.1% of all men were AUDIT positive: 2.9% had AUDIT scores consistent with hazardous drinking, 0.7% with harmful drinking and 0.5% with dependent drinking. In the facility study, 39.6% of men had consumed alcohol in the past year and 5.7% of all men were AUDIT positive: 4.5% had AUDIT scores consistent with hazardous drinking, 0.6% with harmful drinking and 0.6% with dependent drinking.

### Characteristics of men who drink alcohol

The socio-demographic and health-related correlates of AUDIT score are described in Table 2.

**Table 2:** Socio-demographic and health-related associations with continuous AUDIT score and with AUDIT-positive status among men who drink in Kamuli District, Uganda, 2013

Characteristics	AUDIT score, median (IQR)^a^	AUDIT-positive ≥8 (%)^b^
Category		
Age group, years		
18–29	2.0 (1.0, 4.0)*	9.4*
30–44	3.0 (1.0, 5.5)	22.9
45–59	3.5 (2.0, 7.0)	27.5
≥ 60	3.0 (2.0, 6.0)	17.4
Education attainment		
Less the primary education/illiterate	2.0 (1.0, 4.0)	25.6
Completed primary education	3.0 (1.0, 6.0)	21.9
Completed secondary education	3.0 (2.0, 5.0)	17.7
Completed university diploma or degree	4.0 (2.0, 6.0)	16.7
Employment status		
Paid or self-employment	3.0 (2.0, 5.0)*	21.6
Unemployed or volunteer	3.0 (2.0, 7.0)	19.7
Student	1.5 (1.0, 3.5)	0.0
Retired	3.0 (2.0, 5.0)	29.1
Other	1.5 (1.0, 4.0)	0.0
Depression severity		
None	3.0 (1.0, 5.0)*	17.2*
Mild	3.5 (2.0, 7.0)	30.5
Moderate	3.0 (1.0, 7.0)	30.6
Severe	17.0 (11.0, 23.0)	83.3

Combined sample is unadjusted for the survey design features

*AUDIT* Alcohol use disorders identification test

* P < 0.05 for association across all strata

^a^P value calculated using Kruskal–Wallis test

^b^P value calculated using Fischer’s Exact test

There was evidence of association between age and AUDIT score. Men aged (45–59) years had a median AUDIT score of 3.5 (IQR 2, 7.0) where as men aged (18–29) years had a median AUDIT score of 2 (IQR 1, 4.0). The study also found an association between employment status, depression severity and AUDIT score.

In the binary analysis, there was an association between age, depression severity and AUDIT-positive status score. Men aged (45–59) years had the highest AUDIT-positive status score (27.5%) while only 9.4% of men aged (18–29) years had a positive AUDIT score. Men with severe depression had the highest AUDIT-positive where over 8 in every 10 men (83.3%) had a positive AUDIT score. Men with no-depression had the least AUDIT-positive status scores (17.2%).

### Internalized stigma beliefs among AUDIT positive men in the Community Survey

The internalised stigma beliefs among the 17 AUDIT-positive men in the Community Survey are given in Table 3.

**Table 3:** Internalized stigma beliefs among AUDIT-positive men in the PRIME Community Survey (n = 17), Kamuli District, Uganda 2013

Statement	Agree or strongly agree, %
These problems have spoiled my life	47.5
Because of these problems, I need others to make decisions for me	47.0
I feel out of place in the world because of these problems	43.5
People discriminate against me because of these problems	43.1
I am embarrassed or ashamed of these problems	42.5
I am disappointed in myself due to these problems	40.7
I can’t contribute anything to society because of these problems	38.9
People take me less seriously	38.0
Others think that I can’t achieve much in life because of these problems	37.2
Nobody would be interested in getting close to me because of these problems	28.4
People often patronize me or treat me like a child because of these problems	24.2

Figures are adjusted for the population-based sampling design features

Almost half (47.5%) of the AUDIT-positive men reported that alcohol use has ruined their lives while 47% agreed that they need others to make decisions for them. More than four in every 10 (43.0%) men agreed to feeling out of place because of their alcohol use problems. Four out of every 10 men (42.5%) felt embarrassed or ashamed in themselves due to alcohol problems and 28.4% were afraid of discrimination by their loved ones.

### Attitudes and beliefs towards help seeking among AUDIT-positive men in the facility survey

The attitudes and beliefs towards help seeking among the 20 AUDIT-positive men in the facility survey are given in Table 4.

**Table 4:** Attitudes and beliefs among AUDIT-positive men (n = 20) in the PRIME Facility Detection Survey, Kamuli District, Uganda, 2013

Statement	Agree or strongly agree, %
I did not think this was a problem that could be treated	55.0
I thought the problem would get better by itself	40.0
The problem didn’t bother me very much	35.0
I was unsure about where to go or who to see for professional help	25.0
I thought getting professional help would take too much time or be inconvenient	10.0
I wanted to handle the problem on my own	10.0
I had problems with things like childcare or scheduling that would have made it hard to get to treatment	10.0
I felt ashamed or embarrassed to seek professional help for this problem	10.0
I was concerned about how much money it would cost to get professional help	5.0
I was concerned about what others might think if they found out I was seeking professional help	5.0
I was scared about being put into a hospital against my will	5.0
I was not satisfied with available services	5.0
I was concerned that people would think less of me if they found out I am seeking professional help for this problem	5.0
I was concerned that people might treat me differently if they found out that I am seeking professional help for this problem	5.0
I received treatment before and it did not work	0.0
I had problems with things like transportation that would have made it hard to get to treatment	0.0
I was concerned that it might harm my chances of finding or keeping work if people found out I am seeking professional help for this problem	0.0
I was concerned that my family members would not approve	0.0
I was concerned that it might harm my own or my family members’ chances of getting married if people found out I am seeking professional help for this problem	0.0
I was concerned about the treatment I would get, for example, about possible side effects	0.0

In the study, none of the men had sought care for alcohol problems. The majority (55.0%) of the men did not seek care because they did not think alcohol use disorders could be treated. In the study, 4 out of every 10 men did not seek care because they thought they would get better without treatment while (35.0%) were not bothered or inconvenienced by their alcohol use. A quarter (25.0%) of the men did not seek health care because they were unsure about where to go or who to see for professional help. A few (10.0%) of the respondents reported that they were embarrassed to seek care for alcohol use effects and (5.0%) did not seek help because they were not satisfied with available services. Further, 5% of the men did not seek care due to concern of treatment cost.

## Discussion

We found that 21.8% of men in the community survey and 39.6% of men in the facility survey consumed alcohol in the past 1 year. Of those who consumed alcohol in the community survey, 4.1% were AUDIT-positive and 5.8% of those who consumed alcohol in the Facility Survey had behaviours consistent with AUD. AUDIT score was higher among older men as well as men with paid/self-employment status and higher PHQ-9 score. AUDIT-positive men were likely to have internalized stigma and the majority never sought professional treatment, primarily because they did not think AUD could be treated.

The PRIME Facility and Community Survey is the first study of its kind to be conducted in Kamuli district. At 21.8%, the 12 month proportion of men who drink alcohol in the Community Survey was found to be lower compared to other studies done in general community settings in East Africa [29] and in Uganda [1]. Despite the low prevalence of AUD in Kamuli district, the associated detrimental social, economic and health consequences make it an issue of public health priority. Previous Studies conducted in LMICs stipulate that the damaging consequences of alcohol use in low-income countries, start at lower levels of alcohol and affect a significant proportion of the population [30]. In this study, we observe that the people who drink more are likely to have severer depression. Depression among alcohol drinkers is associated with medical non-compliance [31], progressive alcohol dependence [32], violence-related injuries [33], and suicide [34]. We also observed that people who drink more have internalized stigma beliefs. Internalized stigma among people with alcohol use problems is associated with psychiatric symptom severity [35], non-disclosure of mental illness [36] and treatment non-adherence [35, 36]. Other studies in Uganda have also associated alcohol use with an increased risk of HIV infection [14, 37, 38], risky sexual behaviours [38], road traffic accidents [39], sexual coercion [16], poverty persistence [17, 19–21] and intimate partner violence [14, 40]. These notable associations could contribute to the persistently high human poverty indicator in Kamuli currently estimated at 58% [18], and could contribute to the high prevalence of intimate partner violence and HIV in rural eastern Uganda currently estimated at 53% [22] and 6.2% [11], respectively. Further research is required to confirm the actual population-level impact of alcohol use in rural Uganda, findings which will support development of the public health agenda. These findings also provide impetus for the government of Uganda to implement effective policies to reduce the public health impact of alcohol.

In this analysis, 4.1% of men in the Community Survey and 5.8% of men in the Facility Survey screened positive on the AUDIT. Previous community-based [40, 41] and facility-based [12, 38] studies of alcohol in Uganda have found higher percentages of AUDIT-positive score. The lower percentage of AUDIT-positive men in the Community Survey in comparison to other population-based studies may be due to a number of socio-cultural factors including rural residence and availability of alcohol. Previous studies in LMICs report that alcohol distributors primarily target urban areas [1]. This is likely to influence prevalence of AUD in predominantly rural areas like Kamuli, through reduced availability of alcohol. In addition, this study was based on self-reported data, as such, individual’s unwillingness to acknowledge that they drink or have problems with alcohol due to social desirability may have biased our overall prevalence estimates, and stratum-specific prevalence estimates, downwards. The lower prevalence of alcohol use disorders in this study could also be due to the fact that the screening tool (AUDIT), is not validated in the local language used (Luganda). Its Cronbach’s alpha was average; therefore, it’s possible that the AUDIT did not accurately capture the severity of AUD in this population. There is need to locally validate the AUDIT. The AUDIT has been shown to provide an accurate measure of risk across gender, age, and cultures in several LMICs [42, 43]. Having a validated screening tool will enhance opportunistic screening in primary health care settings; consequently narrowing the detection and treatment gap.

In the study, AUDIT-positive scores were highest among adult males aged 45–59 years and least among men aged 18–29 years. These findings are consistent with findings from other studies in Uganda [12, 44, 45] which ascertain that alcohol consumption in the communities is higher among older men. However, they distinctively differ from numerous studies which reported that alcohol use is higher among the younger age group of 18–29 years [10, 15, 38]. The lower prevalence of AUDIT-positive scores among the younger age group may be due to the social desirability bias. Since we used a self-reported questionnaire, the youth unwillingness to acknowledge that they drink or have specific problems with alcohol may likely have biased the prevalence estimates in the younger age group.” A longitudinal survey by Caetano et al. in USA ascertains that younger men are more likely to be non-respondents in alcohol studies, and are also more likely to under-report their drinking behaviour [46]. The lower prevalence of AUD among the younger age group could also be a result of sampling bias. Data collection in the community survey was done at home during working workers. As such, our sample may have captured more young unemployed men who are less likely to afford alcohol. Previous studies on alcohol [47, 48] have cited affordability as one of the most important predictors of alcohol use problems in a population. There is need for qualitative research to better understand the reasons underlying the lower prevalence of AUDIT-positive scores among the younger age group. This will enhance development of age appropriate screening and prevention strategies targeted towards individuals within the highest risk age group.

In the study, there was an association between employment status and AUDIT score. A range of studies ascertain that occupation and work organization conditions play an important role in alcohol intake [49–52] and more broadly, in the problematic use of alcohol as a form of self-medication [52, 53]. Occupation factors such as overtime, lack of intrinsic work rewards, ambiguity about job future have been cited as main factors for heavy and problem drinking [54]. The study findings align with various international studies which ascertain an association between employment status and alcohol use [49, 50, 53, 55, 56]. The findings are also in line with previous studies in Uganda which reported that problems with alcohol were prevalent among people with paid employment [13, 41, 44]. According to Naamara et al., alcohol purchase and consumption is used as a display of economic means in the African culture [44]. Therefore, men with paid employment are more likely to have problems with alcohol because they have the financial means to purchase alcohol both frequently and in larger quantities. However, there is need for further investigation to assess the difference in proportion of income spent on alcohol. This will provide clarity on the effect of alcohol use on standard of living and expenditure on basic needs.

The study found an association between the PHQ-9 scores and AUDIT-positive status scores where men with severe depression had the highest AUDIT-positive status scores. The findings align with other co-morbidity studies in Sub-Saharan Africa and the Western countries which indicate that individuals who suffer from depressive disorders are more likely to have problems with alcohol [2, 13, 57, 58]. According to Martinez et al., causation between AUD and depression is bidirectional [13]: depression increases one’s risk of Alcohol Use Disorders and/or alternatively, problems with alcohol increase one’s risk of depression. This short time relief attained while under intoxication is presumed to lead to a recurrent pattern of alcohol use and subsequently alcohol dependency [2, 6]. However, our findings are contrary with the study conducted in 2008 in southwestern Uganda that did not detect an association between AUDIT scores and depression [13].

Given the increased likelihood of AUD among elderly men and men with depressive disorders, Ministry of Health officials and mental health professionals should design Intervention strategies targeted at these particular high-risk subgroups of the drinking population so as to reduce the public health burden of alcohol misuse. A cost-effectiveness analysis by Chisholm et el. indicates that intervention strategies targeted at particular subgroups of the drinking population are more cost effective for populations with low rates of hazardous drinking than population-wide strategies like taxation [59, 60]. Public health programs should take proactive measures to prevent depressive disorders through addressing the modifiable risk factor of alcohol consumption including providing current information on the risk of alcohol consumption among men and advocating for proper regulation of alcohol sale through more effective alcohol policies.

The study found that AUDIT-positive men endorsed several internalized stigma beliefs. These findings are consistent with those in other studies conducted in Uganda [8, 12, 17, 61] and in other parts of the world [28, 62, 63]. All these studies ascertain that people with AUD do not believe that they are able to make any significant decisions for themselves, and often need others to make decisions for them. Although alcohol is often consumed in groups, the acute withdrawal symptomatology e.g. hangover is often experienced in solitude leaving a sense of loneliness, self-reflection and subsequently feelings of unworthiness. Self-stigma increases secrecy and impedes the seeking of help for alcoholism and in this way, prolongs or aggravates the course of the disease [63]. This informs mental health professionals in primary health care settings, Ministry of Health officials and clinical practitioners of the need to validate the Internalized Stigma of Mental Illness scale for use in routine clinical practice. Having a validated measure of internalized stigma has proven to encourage clinicians in LMICs to include stigma reduction as a verifiable treatment goal in addition to symptom reduction [36]. Given the need for and the low level of uptake of services, it may be useful to consider the role of primary health workers in implementing screening and brief intervention programmes.

In the study, we found that none of the AUDIT-positive men had ever received treatment. They had negative attitudes and beliefs towards seeking professional help and the majority (55.5%) did not know that alcohol use disorders are treatable. This is in line with the situational analysis report which indicated that people in Kamuli are generally unaware of the availability of mental health services in the primary health care centres [8, 17]. The findings are also similar to data reported in other countries [9, 28, 29]. The low continuum of professional help seeking for problems with alcohol could be attributed to the enrooted belief that mental disorders including problems with alcohol are a result of witchcraft [8] plus disbelief in the efficacy of modern medicine in treatment of Alcohol Use Disorders. The belief that detoxification has a series of side-effects such as mental retardation [8] also fuels the inability to seek professional help.

The fact that very few men visit clinics to seek professional care for AUDs accentuates the need for change from clinical to public health (i.e. community-level) practice so as to boost demand for AUD treatment. And will require training of community based health workers in delivery of Screening and Brief interventions (SBI) for AUD. There is great consensus that AUDs can be effectively managed with the existing, limited health care resources available in LMICs by incorporating screening and brief interventions (SBI) within the normal clinical routine of primary health care [64]. Community-level practice will enhance demand and uptake of AUD treatment. The link between internalized stigma and failure to seek professional care suggests that clinicians and District Health officials should integrate stigma reduction into public health efforts to promote alcohol treatment. In addition, personal empowerment interventions should be integrated in AUD treatment programmes as an effective way of reducing internalized stigma. Studies in LMICs have associated personal empowerment with high self-esteem, better quality of life, increased social support, increased satisfaction with mutual-help programs [65, 66] and overall reduction of self-stigmatization [36]. Future studies need to explore if men who do not seek treatment have special needs which are not met in primary health care settings. In addition, a future study on explanatory models is an appropriate means to identify the full range of negative help seeking beliefs and the means to overcome them.

Radio talk shows and community dialogues should be held for mass sensitization on AUDs and the public health burden of alcohol use. In addition, research dissemination activities at district and national level should be endorsed to inform key stakeholders on the need to prioritize internalized-stigma reduction in any efforts to increase uptake for AUD treatment.

There are some important limitations to consider. First, the study is based on self-reported data, individual’s unwillingness to acknowledge that they drink or have problems with alcohol due to social desirability may have biased our overall prevalence estimates, and stratum-specific prevalence estimates, downwards. Second, the screening tool (AUDIT), is not validated in the local language used in Kamuli (Luganda). Its Cronbach’s alpha was average; therefore, it’s possible that the AUDIT did not accurately capture the severity of AUD in this population.

## Conclusion

Internalized stigma beliefs among AUDIT-positive men impede treatment-seeking. Health promotion services informed by commonly expressed internalised stigma beliefs could be an effective step in closing the detection and treatment gap for AUDs. In addition, as part of any efforts to increase detection and treatment services for alcohol use problems, routine Screening and Brief Interventions (SBI) for internalized stigma must be incorporated within the normal clinical routine of primary health care.

## Additional file

**Additional file 1: Table A1.** Locally translated AUDIT.
