# Supplementary material for: Alcohol use in a rural district in Uganda: findings from community-based and facility-based cross-sectional studies
Source: Int J Ment Health Syst. 2018 Apr 3;12:12. doi: 10.1186/s13033-018-0191-5 (PMC5883606; doi:10.1186/s13033-018-0191-5)
Supplement: Supplementary file 1 — Additional file 1: Table A1. Locally translated AUDIT. [file 13033_2018_191_MOESM1_ESM.docx]

N.B Circle all that apply

| A. Basic socio-demographic information | | | | |
| --- | --- | --- | --- | --- |
|  | [Record start time] | HH : MM | | **T0** |
|  | Olina emyaka emeka | 🖎 emyaka | | **AGE** |
|  | [Interviewee sex] | Mwami | 0 | **SEX** |
|  |  | Mukyala | 1 |  |
|  | Wakoma kuddala ki mukusoma? | Sasooma | 1 | **EDU** |
|  |  | Primary school | 2 |  |
|  |  | Secondary school | 3 |  |
|  |  | College/University | 4 |  |
| **A5** | Oyimiridde otya muby’emirimu? | Bankozesa/Nekozesa | 1 | **EMP**  **EMPOT** |
|  |  | Nkola murimu gwa nakyeewa | 2 |  |
|  |  | Sirina murimu | 3 |  |
|  |  | Ndi muyizzi | 4 |  |
|  |  | Nawumula | 5 |  |
|  |  | Ebirala (nyonyola)_________________ | 77 |  |

| B. Screening toolsAUDIT and treatment | | | | | | | | | |
| --- | --- | --- | --- | --- | --- | --- | --- | --- | --- |
| Ngenda kubuuza ebibuuzo ku nkozesa y’omwenge mumwaka oguyise.  [nyonyola ki kyetutegeeza nekigambo ‘mwenge’ ng’owa ebyokulabirako nga biya, waragi, tonto, enguuli nebirala] | | | | | | | | |  |
|  | Mirundi emeeka gy’onywa omwenge? | | | Sinywa [🡪 go to B9] | | | | 0 | **AUD1** |
|  |  |  |  | Mwezi, oba obutawera | | | | 1 |  |
|  |  |  |  | Emirundi wakati wa 2-4 omwezi. | | | | 2 |  |
|  |  |  |  | Emirundi wakati wa 2-3 mu wiiki. | | | | 3 |  |
|  |  |  |  | Mirundi 4 oba egisingawo mu wiiki. | | | | 4 |  |
|  | Olunnaku lw’onywedde obulungi, otera kunywa eby’okunywa bimeeka omuli omwenge? | | | 1-2 | | | | 0 | **AUD2** |
|  |  |  |  | 3-4 | | | | 1 |  |
|  |  |  |  | 5-6 | | | | 2 |  |
|  |  |  |  | 7-9 | | | | 3 |  |
|  |  |  |  | 10 oba egisingawo | | | | 4 |  |
|  | Mirundi emeeka gy’onywa eccupa za beer oba tonto oba waragi oba ekitamiza ekirala kyonna eziweera omukaga oba okusingawo? | | | Sikikolangako | | | | 0 | **AUD3** |
|  |  |  |  | Siweza mwezi | | | | 1 |  |
|  |  |  |  | Mwezi | | | | 2 |  |
|  |  |  |  | Buli wiki | | | | 3 |  |
|  |  |  |  | Buli lunaku oba kyenkana buli lunaku | | | | 4 |  |
|  | ***[ebibuuzo namba B2 ne B3 bwebiba bifunye 0, awo genda butereevu ku B9.]*** | | | | | | | |  |
|  | Mumwaka nga gumu emabeega wali wesanzeko nga tosobola kulekerawo kunywa omwenge kasita oba nga otandiiseko? | | Sikikola ngako | | | 0 | | | **AUD4** |
|  |  |  | Wansi wo mwezi | | | 1 | | |  |
|  |  |  | Mwezi | | | 2 | | |  |
|  |  |  | Buli wiki | | | 3 | | |  |
|  |  |  | Buli lunaku oba kyenkana buli lunaku. | | | 4 | | |  |
|  | Mumwaka oguyise, mirundi emeka gyewesanze nga tosobola kukola bikusubirwamu ku lwo kunywakwo? | | Sikikolangako | | | 0 | | | **AUD5** |
|  |  |  | Wansi wo mwezi | | | 1 | | |  |
|  |  |  | Mwezi | | | 2 | | |  |
|  |  |  | Buli wiki | | | 3 | | |  |
|  |  |  | Buli lunako oba kyenkana buli lunaku. | | | 4 | | |  |
|  | Mumwaka oguyise mirundi emeka gyewesanze nga wetaaga okunywamu kumakya osobole okugenda mumaso n’ebyokola oluvanyuma lw’okunywa ennyo? | | Sikikola ngako | | | 0 | | | **AUD6** |
|  |  |  | Wansi wo mwezi | | | 1 | | |  |
|  |  |  | Mwezi | | | 2 | | |  |
|  |  |  | Buli wiki | | | 3 | | |  |
|  |  |  | Buli lunaku oba kyenkana buli lunaku. | | | 4 | | |  |
|  | Mirundi emeka mumwaka oguyise gye wali ofunnyemu okwejjusa oba okuwulira obuswavu oluvanyuma lw’okunywa? | | Sikikola ngako | | | 0 | | | **AUD7** |
|  |  |  | Wansi wo mwezi | | | 1 | | |  |
|  |  |  | Mwezi | | | 2 | | |  |
|  |  |  | Buli wiki | | | 3 | | |  |
|  |  |  | Buli lunaku oba kyenkana buli lunaku. | | | 4 | | |  |
|  | Mumwaka oguyise mirundi emeka gy’otasobodde kujukira bibaddewo ekiro oluvanyuma lw’okunywa omwenge? | | Sikikola ngako | | | 0 | | | **AUD8** |
|  |  |  | Wansi wo mwezi | | | 1 | | |  |
|  |  |  | Mwezi | | | 2 | | |  |
|  |  |  | Buli wiki | | | 3 | | |  |
|  |  |  | Buli lunaku oba kyenkana buli lunaku. | | | 4 | | |  |
|  | Gwe oba omuntu omulala mwali mufunye ebisago kumibiri oluvanyuma lw’okunywa omwenge? | | Nedda  Yee, naye simumwaka oguwedde  Yee, mumwaka oguwedde | | | 0 | | | **AUD9** |
|  |  |  |  |  |  | 2 | | |  |
|  |  |  |  |  |  | 4 | | |  |
|  | Waliwo ow’oluganda lwo oba mukwano gwo oba omusawo eyali alaze okufayo mungeri gy’onywamu omwenge nakusaba okendezeko? | | Nedda | | | 0 | | | **AUD10** |
|  |  |  | Yee, naye simumwaka oguwedde | | | 2 | | |  |
|  |  |  | Yee, mumwaka oguwedde. | | | 4 | | |  |
|  | Total score for B1-B10 | | 🖎________ | | |  | | | **AUDTOT** |
|  | AUDIT score (=B11) | | <8 [AUDIT-POSITIVE] | | |  | | |  |
|  |  |  | ≥8 [AUDIT-NEGATIVE] | | |  | | |  |
| **AUD Internalized Stigma** | | | | | | | | | |
| Ogambye nti mumwaka oguyise, wafuna obuzibu obutali bumu ngabuva kukunywakwo okw’omwenge. Ngenda kubuuza ebibuuzo ebikwatagana kubuzibu bunno. Mbulira oba okiriziganya, oba tokiriziganya nebino wamanga. | | | | | | | AUDST_ | | |
| SA1 | | Olwebizibu bino, mpulira nga sirina mirembe munsi muno | | | Sibwekiri yadde akatono  Sibwekiri  Bwekiri  Bwekiri dala | 1  2  3  4 | _ISMI01 | | |
| SA2 | | Mpulira obuswavu olw’ebizibu bino | | | Sibwekiri yadde akatono  Sibwekiri  Bwekiri  Bwekiri dala | 1  2  3  4 | _ISMI05 | | |
| SA3 | | Mpulira nga n’amaanyi gampedemu olwebizibu bino | | | Sibwekiri yadde akatono  Sibwekiri  Bwekiri  Bwekiri dala | 1  2  3  4 | _ISMI16 | | |
| SA4 | | Ebizibu bino binyononedde obulamu bwange | | | Sibwekiri yadde akatono  Sibwekiri  Bwekiri  Bwekiri dala | 1  2  3  4 | _ISMI17 | | |
| SA5 | | Olwebizibu bino, sikyasobola kwesalirawo. Netaaga obuyambi bwabalala okusobola okusalawo | | | Sibwekiri yadde akatono  Sibwekiri  Bwekiri  Bwekiri dala | 1  2  3  4 | _ISMI19 | | |
| SA6 | | Sikyalina nakyenyinza kukolera nsiyange, olwebizibu bino | | | Sibwekiri yadde akatono  Sibwekiri  Bwekiri  Bwekiri dala | 1  2  3  4 | _ISMI23 | | |
| SA7 | | Abantu bansosola olwebizibu bino | | | Sibwekiri yadde akatono  Sibwekiri  Bwekiri  Bwekiri dala | 1  2  3  4 | _ISMI03 | | |
| SA8 | | Abantu ebiseera ebisinga bampisa ngamwana muto olwebizibu bino | | | Sibwekiri yadde akatono  Sibwekiri  Bwekiri  Bwekiri dala | 1  2  3  4 | _ISMI15 | | |
| SA9 | | Abantu tebandabawo, era tebantwala ng’ensonga olwebizubu bino | | | Sibwekiri yadde akatono  Sibwekiri  Bwekiri  Bwekiri dala | 1  2  3  4 | _ISMI22 | | |
| SA10 | | Teri muntu yandyetaaze okuninaana olwebizibu bino | | | Sibwekiri yadde akatono  Sibwekiri  Bwekiri  Bwekiri dala | 1  2  3  4 | _ISMI25 | | |
| SA11 | | Abalala balowooza nti sirina kyamaanyi kyenyinza kutuukako olwebizibu bino. | | | Sibwekiri yadde akatono  Sibwekiri  Bwekiri  Bwekiri dala | 1  2  3  4 | _ISMI28 | | |
|  | | | | | | | | | |

|  | Mumyezi e 12 egiyise emabeega wali oyogeddeko n’omuntu yenna ng’olaga okweralikirira olwokunywa kwo? | Nedda[🡪go to B14] | 0 | **AUDDISC** |
| --- | --- | --- | --- | --- |
|  |  | Yee | 1 |  |
|  | Ani gwewayogera naye?  Waliwo Omuntu omulala? | Ow’omukwano oba mulirwana wange | 1 | **AUDDISC_**  **FRIEND**  **SPOUSE**  **OFAM**  **EMPL**  **REL**  **HCWORK OTHER** |
|  |  | Muganzi wange | 2 |  |
|  |  | Ow’oluganda wo omulala yenna | 3 |  |
|  |  | Mukama wange/ omukozi gwe nkola naye | 4 |  |
|  |  | Munaddini | 5 |  |
|  |  | owebyobulamu (okugeza nga omusawo wekinansi, dokita/nurse, omukugu mubyomwomwenge) | 6 |  |
|  |  | Omulala (specify)  🖎 | 77 |  |
|  | Mumyezi ekumi n’ebiri egiyise, wali ofunyeko obujjanjabi olw’okunywa omwenge? | Nedda[🡪go to B32] | 0 | **AUDTX** |
|  |  | Yee | 1 |  |
|  |  | simanyi[🡪go to B32] | 888 |  |
|  | Obujjanjabi wabufuna kuva wa mukugu ani?  [Chose all that apply before continuing.  Complete both sections from B16 and B24 if necessary.]  Waliwo ebalala? | *Dokita omukugu/kakensa* Psychiatrist [🡪go to B16] | 1 | **AUDTX_**  **_PSY**  **_OSPEC**  **_GENDOC**  **_OGEN**  **_REL**  **_TRAD**  **_OTHER** |
|  |  | *Omukugu omulala munddwadde z’emitwe; nga kansala* [🡪go to B16] | 2 |  |
|  |  | *Dokita owabulijjo* [🡪go to B24] | 3 |  |
|  |  | *Omusawo ogugeza omusawo wo kukyalo*, nurse[🡪go to B24] | 4 |  |
|  |  | Munadini[🡪go to B32] | 5 |  |
|  |  | Omusawo wekinansi[🡪go to B32] | 6 |  |
|  |  | Omulala [specify]  🖎  [🡪go to B32] | 66 |  |
